# Reference genes for gene expression studies by RT-qPCR in *Brevipalpus yothersi* (Acari: Tenuipalpidae), the mite vector of citrus leprosis virus

**DOI:** 10.1038/s41598-019-42993-2

**Published:** 2019-04-25

**Authors:** Luana Aparecida Rogerio, Diogo Manzano Galdeano, Gabriella Dias Arena, Maria Andreia Nunes, Marcos Antonio Machado, Valdenice Moreira Novelli

**Affiliations:** Sylvio Moreira Citrus Center, Agronomic Institute (IAC), Cordeirópolis, São Paulo Brazil

**Keywords:** Gene expression, RNAi, Gene expression profiling

## Abstract

Quantitative reverse transcription PCR (RT-qPCR) is a high-throughput method to analyze the transcriptional expression of genes. Currently, no reference genes have been described for evaluating gene expression in *Brevipalpus yothersi*, the false spider mite, a polyphagous that act as vector of the citrus leprosis virus C (CiLV-C), an important citrus disease. This study aimed to identify the most stable reference genes in *B. yothersi*. The RT-qPCR expression data for selected genes were evaluated from three conditions: different developmental stages, plant hosts and acquisition of CiLV-C. To analyze the stability of the candidate reference genes we used ΔCq method, GeNorm, NormFinder, BestKeeper and RefFinder. *Ubiq* and *GAPDH* are best suited for normalizing gene expression data in viruliferous and non-viruliferous mites. *Ubiq*, *EF1α* and *GAPDH* are the most stable for different developmental stages. *RPL13* and *RPL32* are the best reference genes for approaches to *B. yothersi* in different host plants. Considering all the experimental conditions, *Ubiq*, *EF1α*, and *GAPDH* were the most stable genes. Here we developed an accurate and comprehensive RT-qPCR strategy for use in *B. yothersi* gene expression analysis. These results will improve the understanding of the biology of the false spider mites and their role as virus vectors.

## Introduction

The false spider mites *Brevipalpus* spp. (Acari: Tenuipalpidae) are phytophagous, polyphagous, worldwide distributed, and considered one of the most prominence agricultural pests by association with more than 40 plant viruses. The so-called *Brevipalpus*-transmitted viruses (BTVs) severely affect a wide range of plant species, including economically important ones such as citrus, coffee, passion fruit, and orchids^1^. Within the nearly three hundred species of the genus, *Brevipalpus yothersi* is attributed as the main vector of the citrus leprosis virus C (CiLV-C), the causal agent of a common and damaging citrus disease in the Americas^2–4^. Citrus leprosis disease threatens the citrus orchards affecting fruit quality, leading to severe yield losses and even the plant death, with consequent increase in the production costs^5^. In Brazil, the management of citrus leprosis is performed mainly by the chemical control of the mite vector, with annual estimated costs of US$ 55 million/^6^. Worldwide, the control of the false spider mite accounts for 10% of the total acaricide market^7^. In addition to the financial cost, the widespread use of acaricides represent a threat to the environment with the development of resistant mite populations as well as soil and water contamination.

Besides this economic importance, *B. yothersi* is also biologically interesting due to the representation of mite populations entirely by haploid females (n = 2 chromosomes) with thelytokous reproduction. This phenomenon is attributed to the *Cardinium* symbiont, which feminize unfertilized eggs resulting on highest number of females while the males comprises only up to 3,3% of the natural populations. These features make the false spider mites completely unusual organisms in Metazoan^8^.

Although previous studies reported aspects of the biology of the false spider mites^9,10^ and its interactions with plant hosts^11–14^, to the best of our knowledge no information of functional genomics in vector-virus interactions is available, additionally, first draft of the *B. yothersi* genome was published bring up genes to be elucidated^15^. In this context, comparative transcriptome profile has been showed as a powerful tool for mite studies^16–18^. A better understanding of the biology and interactions of the false spider mite as virus vector and functional genomic analysis under different conditions are desirable to stablish further strategies of control of *Brevipalpus*-causing diseases.

Reverse transcription quantitative polymerase chain reaction (RT-qPCR) is a rapid and reliable method for the detection and quantification of changes in gene expression under different biological process. However, the use of RT-qPCR approach requires the control of unspecific variations between samples such as those caused by differences in pipetting, RNA quantification and integrity, or reverse transcriptase efficiency^19^. This control is achieved by using genes with constant expression independent of the sample physiological condition and the applied treatment^20^, referred to as reference genes. Designing an appropriate strategy to normalize the expression of target genes with the most stable reference genes is vital to enhance the reliability and reproducibility of RT-qPCR^21^.

Housekeeping genes including *elongation factor 1 α* (*EF1α*), *glyceraldehyde-3-phosphate dehydrogenase* (*GAPDH*), *ribosomal protein L13* (*RPL13*) and *α-tubulin* (*Tub*) have been used as reference genes for the normalization of RT-qPCR data of other phytophagous mites^17,22–24^. However, the expression levels of these reference genes might vary for different mites or environmental conditions, leading to erroneous data interpretation and conclusions. Therefore, before expression studies it is important to stablish and validate stable reference genes according to the organism and to the experimental conditions^20,25^. Here, our goals were to identify suitable reference genes to develop an accurate and comprehensive RT-qPCR method for use in *B. yothersi* gene expression analyses. Seven candidate reference genes were selected from the *B. yothersi* genome: *actin* (*Act*), *EF1α*, *GAPDH*, *RPL13*, *ribosomal protein L32* (*RPL32*), *Tub* and *ubiquitin* (*Ubiq*). The stability and performance of these genes were evaluated in *B. yothersi* mites under different experimental conditions, including different developmental stages, distinct plant hosts and acquisition of CiLV-C.

## Results

### Expression profiles of candidate reference genes

**Figure 1:**
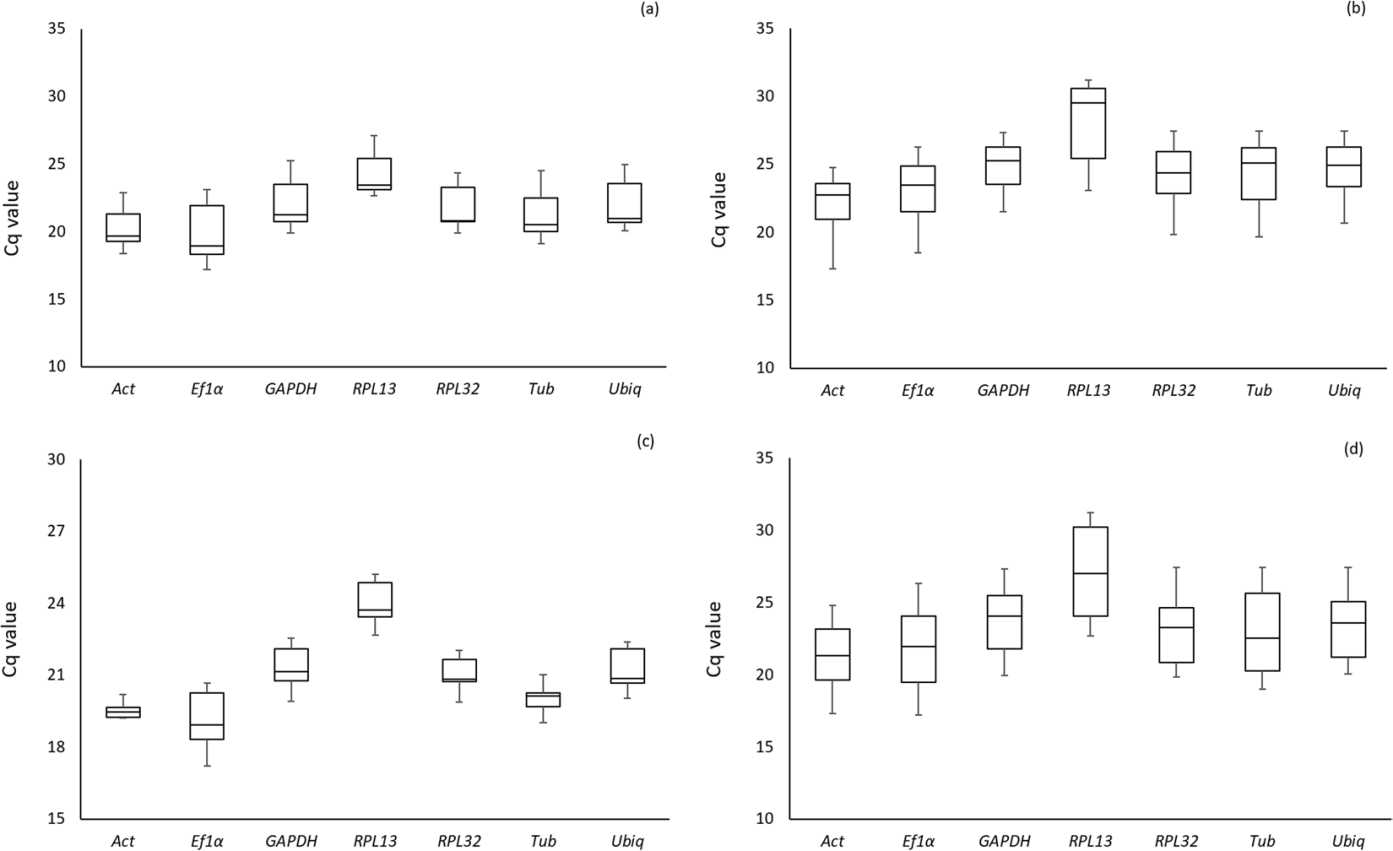
Expression profiles of candidate reference genes of *B. yothersi* from distinct experimental assays. (**a**) CiLV-C viruliferous and non-viruliferous mites, (**b**) mites from different developmental stages, (**c**) mites reared in different plant hosts and (**d**) total mite samples from all conditions. The expression levels in the samples are documented in Cq value. The line in the box represents the median. The interquartile rang is bordered by upper and lower edges, corresponding the 75^th^ and 25^th^ percentiles, respectively. The circle indicates an outlier.

Assays designed to quantify the transcript levels of each candidate reference gene were optimized by PCR and RT-qPCR. All primer pairs were validated by the detection of single amplicons of the expected sizes, ranging from 59 to 128 bp, in a 1.2% agarose gel electrophoresis. The sequencing of these amplicons displayed 100% identity with their corresponding transcripts. Primer specificity was confirmed by the presence of a single-peak in melting curve analysis. Average Cq values ranged from 17.20 (*EF1α*) to 26.96 (*RPL13*) for viruliferous and non-viruliferous mites (Fig. 1a); from 17.29 (*Actin*) to 31.18 (*RPL13*) for mites of different developmental stages (Fig. 1b); from 17.34 (*EF1α*) to 25.12 (*RPL13*) for mites feeding from different host plants (Fig. 1c); and from 17.20 (*EF1α*) to 31.19 (*RPL13*) for all treatments (Fig. 1d).

### Gene expression stability

The expression stability of the candidate reference genes was evaluated using comparative ΔCq method^26^, GeNorm^19^, NormFinder^27^, and BestKeeper^28^.

### CiLV-C viruliferous x non-viruliferous mites

All methods had similar performance using the false spider mites in the CiLV-C assays. *Ubiq* and *GAPDH* were consistently identified as the most stable genes in non-viruliferous and viruliferous mites. In the same set *EF1α*, *Act* and *RPL13* were the least stable according to ΔCq, GeNorm and NormFinder, whilst *RPL32* replaced *Act* as one of the three least stable on the BestKeeper ranking.

### Different developmental stages

Likewise at the comparison of CiLV-C viruliferous x non-viruliferous, the distinct algorithms generated similar results to false spider mites from different developmental stages. *EF1α* and *Ubiq* were the best whilst *Tub* and *RPL13* were the worst reference genes among the candidates.

### *B. yothersi* from different host plants

In samples of mites that were reared in different plant hosts, the gene pairs *RPL13/ RPL32* and *GAPDH/ RPL13* were ranked as the most stable by the ΔCq method and GeNorm, respectively. On the other hand, NormFinder and BestKeeper attributed *GAPDH* and *RPL32* as the genes with highest stability in samples from it same treatment. The least stable genes in false spider mites from different plant hosts were *Tub/EF1α* according to GeNorm and NormFinder, and *Act/Tub* using ΔCq and BestKeeper.

Finally, considering all experimental conditions (virus acquisition, development stages, and plant hosts) in global analysis, distinct algorithms resulting on dissimilar rankings to reference genes. Both ΔCq method and BestKeeper identified *EF1α* as the most stable gene, NormFinder ranked *Ubiq* as the one with lowest variation between all samples, and GeNorm ranked *GAPDH*/*Act* genes as the best between all candidates. In the same way, the least stable genes were *RPL13* to ΔCq and GeNorm, *EF1α* using NormFinder, and *Tub* according BestKeeper results.

### Ranking of best reference genes using RefFinder

The RefFinder tool was used to define the overall final ranking of reference genes (Table 1), by calculating the geometric mean (GM) of the weights attributed to each candidate gene for each software described above^29^. According to RefFinder parameters, the genes *Ubiq* (GM = 1.68) and *RPL13* (GM = 6) were the most and least stable, respectively, when samples from all experimental conditions were analyzed together. In viruliferous and non-viruliferous mites, the stability of expression (from highest to lowest) ranked the genes as follows: *Ubiq* > *GAPDH* > *Tub* > *EF1α* > *RPL13* > *Act* > *RPL32*. To different development stages of the false spider mites, the gene reference order recommended by RefFinder (starting from the best candidate) was: *Ubiq* > *EF1α* > *GAPDH* > *RPL32* > *Act* > *Tub* > *RPL13*. Finally, when mites were reared in different host plants, the genes were ranked from the most to the least stable in the following order: *RPL13* > *RPL32* > *GAPDH* > *Ef1α* > *Ubiq* > *Act* > *Tub*.

### Number of reference genes for normalization by RT-qPCR

**Figure 2:**
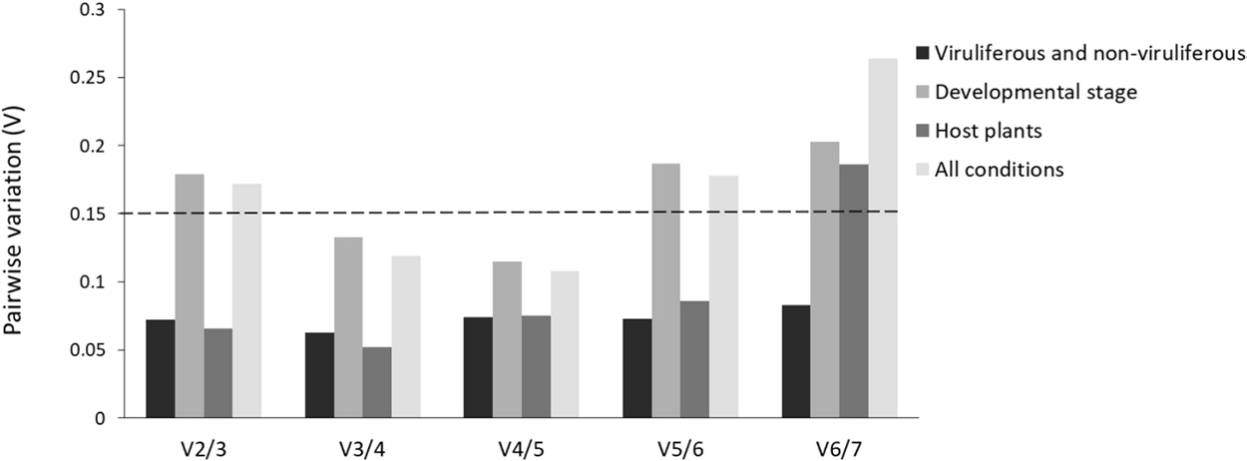
Determination of the optimal number of reference genes for RT-qPCR normalization. The pairwise variation (V_n_/V_n+1_) was analyzed for normalization factors NF_n_ and NF_n+1_ by GeNorm software to determine the optimal number of references genes required for RT-qPCR data normalization. Values < 0.15 indicate that additional genes are not required for the normalization of gene expression.

For robust and consistent RT-qPCR results, at least two reference genes are required. Thus, the pairwise variation (V_n_/V_n+1_) was performed using GeNorm to determine the ideal number of reference genes for each experimental condition (Fig. 2). The cut-off value of 0.15 was used to indicate the need for inclusion of additional reference genes in a normalization factor^19^. In the experimental assays with non-viruliferous and viruliferous mites, or mites from different plant hosts, the values of V2/3 were lower than 0.15, indicating that two genes are reliable to accurate normalization of RT-qPCR. On the other hand, in false spider mites from different developmental stages, or samples from all conditions merged, the V2/3 parameter were 0.179 and 0.186, respectively, indicating that the addition of a third reference gene would improve the reliability of normalization.

### Validation of reference genes

**Figure 3:**
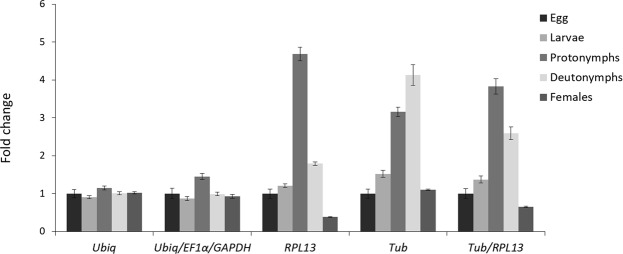
Transcript level of the *V-ATPase* transcription factor gene in different development stages of the *B. yothersi*. Normalization factors were calculated as the geometric mean of the expression levels of the three most stable reference genes (*Ubiq, EF1α* and *GAPDH*) and the two most unstable (*RPL13* and *Tub*). Error bars show average standard error calculated from three biological replicates.

The expression of the *V-type proton ATPase catalytic subunit A* (*V-ATPase*), a false spider mite target gene, was evaluated to validate reference genes from different developmental stages of *B. yothersi*. The *V-ATPase* transcript levels were normalized with the three most stable (*Ubiq*, *EF1α* e *GAPDH*) and the two most unstable (*RPL13* e *Tub*) reference genes (Fig. 3), as determined by RefFinder (Table 1). The normalization of *V-ATPase* expression with the best or the three best reference genes resulted in nearly invariable expression between mite stages. However, when the two worst unstable genes were used, individually or combined as a normalization factor, the *V-ATPase* fold-change increased in larvae, protonymph and deutonymph relative to the egg control (Fig. 3).

**Table 1:** Stability of candidate reference genes under different experimental conditions.

Experimental Conditions	Rank	RefFinder		ΔCq		GeNorm		NormFinder		BestKeeper	
		Gene	GM^a^	Gene	SD^b^	Gene	M^c^	Gene	SV^d^	Gene	r^e^
Viruliferous and Non-viruliferous	1	*Ubiq*	1.86	*Ubiq*	0.51	*Ubiq*	0.18	*Ubiq*	0.05	*GAPDH*	0.99
	2	*GADPH*	2.45	*GADPH*	0.55	*GAPDH*	0.18	*GADPH*	0.06	*Ubiq*	0.99
	3	*Tub*	2.91	*Tub*	0.56	*Tub*	0.23	*Tub*	0.23	*Tub*	0.99
	4	*EF1α*	3.98	*RPL 32*	0.57	*RPL 32*	0.28	*RPL 32*	0.25	*Act*	0.99
	5	*RPL 13*	4.16	*RPL 13*	0.57	*Act*	0.31	*Act*	0.32	*EF1α*	0.99
	6	*Act*	4.3	*EF1α*	0.58	*RPL 13*	0.35	*PLR13*	0.43	*RPL 32*	0.99
	7	*RPL 32*	4.47	*Act*	0.65	*EF1α*	0.41	*EF1α*	0.54	*RPL 13*	0.97
Developmental stage	1	*Ubiq*	1.68	*EF1α*	0.8	*Ubiq*	0.26	*EF1α*	0.16	*EF1α*	0.99
	2	*EF1α*	1.97	*Ubiq*	0.83	*RPL13*	0.26	*Ubiq*	0.44	*Ubiq*	0.98
	3	*GAPDH*	2.45	*GAPDH*	0.89	*EF1α*	0.41	*RPL 32*	0.45	*GAPDH*	0.98
	4	*RPL 32*	2.83	*RPL 32*	0.92	*GAPDH*	0.48	*GADPH*	0.47	*RPL 32*	0.98
	5	*Act*	4.4	*Act*	0.94	*Act*	0.54	*Act*	0.57	*Act*	0.97
	6	*Tub*	6	*Tub*	1.21	*Tub*	0.72	*Tub*	0.97	*RPL 13*	0.95
	7	*RPL 13*	7	*RPL 13*	1.23	*RPL 13*	0.86	*RPL 13*	1.09	*Tub*	0.93
Host plants	1	*RPL 13*	2	*RPL 13*	0.5	*GAPDH*	0.1	*GADPH*	0.05	*GAPDH*	0.99
	2	*RPL 32*	2.51	*RPL 32*	0.54	*RPL 13*	0.1	*RPL 32*	0.09	*RPL 32*	0.99
	3	*GADPH*	2.91	*GADPH*	0.54	*Ubiq*	0.14	*Ubiq*	0.11	*RPL 13*	0.99
	4	*EF1α*	3.64	*Ubiq*	0.54	*RPL 32*	0.19	*RPL 13*	0.13	*EF1α*	0.98
	5	*Ubiq*	3.66	*EF1α*	0.55	*Act*	0.27	*Act*	0.36	*Ubiq*	0.98
	6	*Act*	3.83	*Act*	0.61	*Tub*	0.34	*Tub*	0.5	*Act*	0.93
	7	*Tub*	5.66	*Tub*	0.63	*EF1α*	0.39	*EF1α*	0.52	*Tub*	0.83
All conditions	1	*Ubiq*	1.68	*EF1α*	0.81	*GAPDH*	0.26	*Ubiq*	0.05	*EF1α*	0.99
	2	*EF1α*	1.97	*Ubiq*	0.82	*Act*	0.26	*GADPH*	0.06	*GAPDH*	0.98
	3	*GAPDH*	2.28	*GAPDH*	0.85	*Ubiq*	0.4	*Tub*	0.23	*Ubiq*	0.98
	4	*Act*	3.34	*RPL 32*	0.88	*RPL 32*	0.44	*RPL 32*	0.25	*RPL 32*	0.98
	5	*RPL 32*	4	*Act*	1.01	*EF1α*	0.5	*Act*	0.32	*RPL 13*	0.97
	6	*Tub*	6	*Tub*	1.14	*Tub*	0.69	*RPL 13*	0.43	*Act*	0.97
	7	*RPL 13*	6	*RPL 13*	1.16	*RPL 13*	0.8	*EF1a*	0.54	*Tub*	0.96

^a^GM: geometric mean: ^b^SD standard deviation of comparative ΔCq. ^c^M: average of stability expression values. ^d^SV: Stability value. ^e^R: Pearson’s correlation.

## Discussion

Gene expression analysis is a powerful way to understand the uncommon reproduction system of *Brevipalpus* and the complex interaction between these mites with plants and viruses. Nevertheless, information on the expression of false spider mites genes is scarce, particularly due to the absence of standard protocols. RT-qPCR has been a sensitive, specific and reproducible tool for gene expression analysis^30^. To obtain feasible RT-qPCR measurements, the expression of target genes must be normalized by reference genes with stable expression in the different biological conditions that the target genes will be tested^31,32^.

In the last years, an increasing number of studies has established suitable reference genes for arthropods^33–35^, in case of phytophagous mites, the studies of reference genes includes xenobiotic toxicity, development stage, abiotic stress and shift plant host^22,23,36,37^. *GAPDH* was found as best housekeeping genes for *T. urticae* in case of plant shift studies and *RPL13* was cited one as the best reference genes for development studies for this mite^36^. While for *Panonychus citri*, in development studies and abiotic stress, besides *GADPH*, *EF1α* was the most stable genes^23^. *RPL13* was chosen as best in development stages studies a for *T. cinnabarinus*^22^. Whereas for *Brevipalpus* species there are no reference gene information.

Here, we evaluated the expression stability of seven *B. yothersi* candidate reference genes under different conditions: CiLV-C acquisition (viruliferous and non-viruliferous populations), different developmental stages (eggs, larvae, protonymphs, deutonymphs and adult females), and rearing on distinct host plants (sweet orange and common bean). Our study show that all seven candidates are stable under the tested conditions and can be immediately used for normalization of RT-qPCR studies, because all tested genes exhibited high expression stability with M < 0.86. We also present the best ones for each biological condition and the recommended number of reference genes to obtain reliable results. We used four different algorithms (Comparative ΔCq, NormFinder, GeNorm and BestKeeper) to identify the most stable genes. Despite small differences in their results, as expected^38,39^, similar sets of reference genes were identified for each experimental condition. The RefFinder tool was used to combine the results from the distinct algorithms in a final classification.

The RefFinder classified the *Ubiq* and *GAPDH* genes as the most stable across viruliferous and non-viruliferous false spider mite populations (Table 1). The main function of ubiquitin proteins is the selective degradation of short-lived protein in eukaryotic cells, performing a housekeeping role in the control in numerous cellular process^40^. *GAPDH* encodes an enzyme essential for glycolysis and glycogenesis pathways, among others functions in several cellular processes^41,42^. *Ubiq* gene was previously identified as the most stable candidate gene in *Delphacodes kuscheli* infected with Mal de Río Cuarto virus (MRCV)^43^ and *Bombus terrestres* infected with Israeli acute paralysis virus^44^. At the same viruliferous and non-viruliferous comparison, we identified *Act* and *RPL32* as the least stable genes. Both genes were revealed as the most reliable ones in *Rhopalosphum padi* non-viruliferous and viruliferous for Barley yellow dwarf virus^45^, supporting the notion that gene stability must be checked for each specie and condition. The pairwise variation analysis revealed that two reference genes perform trustfully (V2/3 < 0.15) across mite samples with and without CiLV-C, and hence adding more genes would not increase the confidence of the analysis (Fig. 2). We suggest using *Ubiq* and *GAPDH* in transcriptome studies with viruliferous and non-viruliferous *Brevipalpus* mites.

At *B. yothersi* mites from different development stages the *Ubiq*, *EF1α*, and *GAPDH* genes were identified as the most stable, whilst *Tub* and *RPL13* were the least stable ones. *Ubiq* has been used to normalize RT-qPCR data from different developmental stages of the bettle *Agrilus planipennis*^46^. GAPDH was identified as a reliable reference gene for *Spodoptera litura*^47^ and *Sesamia inferens*^48^ caterpillars, and *Lipaphis erysimi*^35^ thrips. *EF1α*, coding for a protein involved in the delivery of aminoacyl tRNAs to the acceptor site on ribosomes during protein translation^49^, was suggested as a reference gene for *Aphis glycines*^50^, *Diabrotica virgifera virgifera*^51^*, Panonychus citri*^23^, *Locusta migratoria*^52^, *Frankliniella occidentalis*^53^ and *Coleomegilla maculata*^24^. Even though *Tub* and *RPL13* were the least stable in *B. yothersi* mites from different development stages, they were pointed as the most stable ones in *Lipaphis erysimi*^35^, *Tetranychus urticae*^37^ and *T. cinnabarinus*^22^. The pairwise variation analysis indicated the use of at least three reference genes to ensure reliable RT-qPCR analysis (V2/3 > 0.15) (Fig. 2). Thus, we suggest using *Ubiq*, *EF1α*, and *GAPDH* to normalize RT-qPCR data comparing *Brevipalpus* mites from distinct developmental stages.

In RefFinder analyses, we find the *RPL13* and *RPL32* ribosomal protein-coding genes as the most stable genes across *B. yothersi* populations fed on beans or sweet orange fruits. A limited number of studies have tested reference genes from arthropods feeding on distinct host plants or artificial diets. These works identified *EF1a* as the best reference for *Drosophila melanogaster*^31^ and *Lipaphis erysimi*^35^, and *GAPDH* as the most stable in *T. urticae* populations^36^. Nevertheless, ribossomal protein genes have been extensively used for RT-qPCR normalization of data from a wide range of species and conditions^39,48,50,54^. The pairwise variation analysis indicated that the use of two reference genes is enough to ensure the reliability of RT-qPCR normalization (V2/3 < 0.15) in mites reared on beans or sweet orange fruits. Hence, we suggest using *RPL13* and *RPL32* in comparisons of *Brevipalpus* mite populations reared on different host plants.

Combining all the experimental conditions applied to *B. yothersi* mites, *Ubiq*, *EF1α*, and *GAPDH* were classified as the most stably expressed reference genes. The pairwise variation analysis indicated that using three genes (V3/4 < 0.15) improve the data confidence (Fig. 2) when mites samples comprising all sources of variability analyzed here (virus presence, different developmental stages and feeding from distinct host plants) are assayed. However, when the distinct experimental conditions are individually analyzed, we identified different sets of genes as the most stably expressed, reinforcing the idea that there is no universal reference gene for every situation and suggesting the use of the best gene set at each condition.

Finally, to validate the reliability of the reference genes identified here, we evaluated the normalized expression profile of *V-ATPase* gene in different development stages of *B. yothersi*. We found variable gene expression between treatments when we normalize the transcript levels using the most unstable gene (*RPL13*) or a combination of the two most unstable ones (*RPL13*/*TUB*) (Fig. 3). Results using inferior reference genes led to the conclusion that proto or deutonymphs express *V-ATPAse* at a higher level that the other mite phases. On the other hand, using the most stable gene or the three more stables ones combined (*Ubiq/EF1α/GAPDH*) to normalize the RT-qPCR data, no difference in *V-ATPAse* expression was observed between the development stages (Fig. 3). These results indicate that the use of unstable reference genes might mask or introduce artificial changes in gene expression, leading to misinterpretations of biological phenomena. Similar results have been found in RT-qPCR studies from other systems, such as *Drosophila melanogaster*^55^ or citrus plants^56^, emphasizing the need for testing a set of reference genes for each experimental condition assayed.

This study is the first evaluation of the expression stability of *B. yothersi* genes and provides a standardized procedure for gene expression analyses of false spider mites. The results presented here will allow the precise normalization in RT-qPCR studies involving *Brevipalpus* mites gene function and mite/virus interactions and will contribute to improve our understanding of the molecular mechanisms involved in the citrus leprosis and others viral diseases vectored by false spider mites.

## Materials and Methods

### Mite rearing and synchronization

*B. yothersi* isoline has been reared on healthy sweet orange fruits (*Citrus sinensis* L. Osbeck) at 25 ± 5 °C, 60 ± 10% RH and 14:10 h (light: dark) photoperiod conditions since 2008, on at the Acarology Laboratory from the Sylvio Moreira Citrus Center, Agronomic Institute (IAC), Cordeirópolis (São Paulo State, Brazil). To synchronize mite ages, adult females were placed on sweet orange fruits for egg-laying and removed after 24 h.

### Developmental stage

Five different stages (egg, larvae, protonymphs, deutonymphs and adult females) were collected from *B. yothersi* age-synchronized colonies onto healthy sweet orange fruits. Pools of 500 eggs, 400 larvae, 400 protonymphs, 300 deutonymphs, and 300 females were transferred directly to microcentrifuge tubes. To each developmental stage, three replicates were independently collected. The samples were flash frozen in liquid nitrogen and stored at −80 °C until use.

### Mites host

*B. yothersi* mites were reared on plants of sweet orange and common bean (*Phaseolus vulgaris* L.) and maintained at 25 ± 5 °C, 60 ± 10% RH with a photoperiod of 14:10 h (light: dark). After at least three mite generations, pools of 300 females of each host plant were collected in three biological replicates. Samples were flash frozen and stored at −80 °C until use.

### CiLV-C acquisition

Viruliferous samples were obtained by rearing the non-viruliferous false spider mites onto sweet oranges fruits with citrus leprosis symptoms for several generations. Three biological replicates with pools of the 300 viruliferous mites were collected and flash frozen stored at −80 °C. The CiLV-C acquisition was confirmed by RT-PCR using specific primers to detected a region within the viral movement protein (MP) gene, according to methodology described by Locali *et al*.^57^.

### RNA extraction and cDNA synthesis

Total RNA was extracted from each development stage using RNAqueous®-Micro Kit (Ambion, Life Technologies). The RNA concentration was measured with a NanoDrop ND-8000 spectrophotometer (Thermo Scientific) and RNA integrity was checked in 1% agarose gel. RNA samples with an A260/A280 ratio ranging from 1.8 to 2.2 were used for cDNA synthesis. First-strand complementary DNA was synthesized from 200 ng of total RNA with Platus Transcriber RNase H- cDNA First Strand kit (Sinapse Inc, catalog number S1402) following the manufacturer´s instructions and stored at −20 °C until use. The cDNA was diluted ten-fold for the subsequent RT-qPCR analysis.

### Reference gene selection and primer design

Seven commonly reference genes used in arthropod studies were selected as candidates (Table 2). Gene sequences were obtained from the *B. yothersi* database genome^15^. Primers were designed using PrimerQuest tool (https://www.idtdna.com/Primerquest/Home/Index) with default parameters for qPCR and settings to span exon junctions. PCR amplifications were performed in 25 µl reactions using GoTaq® Colorless Master Mix 2 × (Promega), containing 0.5 µl of each primer (10 µM) and 3 µl of cDNA under the following parameters: one cycle of 94 °C for 3 min, 35 cycles of 94 °C for 30 s, 60 °C for 30 s, and 72 °C for 20 s, a final cycle of 72 °C for 10 min. PCR products were visualized in 1.2% agarose gel and purified with QIAquick® PCR Purification kit (Qiagen) for sequencing confirmation using ABI BigDye Terminator Version 3.1 cycle sequencing (Thermo Fisher).

**Table 2:** Gene name and primer information for RT-qPCR assays.

Gene name	Gene Abbreviation	Primer sequence (5′-3′)	Length (pb)	Mean Efficiency (%)*
*Actin*	*Act*	F: CCAGTGGTACGACCAGATGC	59	88.26
		R: CCATGTATGTGGCCATCCAA		
*Ribosomal protein L13*	*RPL13*	F: ATGGAGGTGGCGTATGTATG	128	82.64
		R: ACTGAGTGCCTTGTAAACCG		
*Glyceraldehyde-3-phosphate dehydrogenase*	*GAPDH*	F: ACCTGTCGACTGAAGAAAGATG	114	80.56
		R: TACGGCCTGTTCATCACAATAA		
*Ribosomal protein L32*	*RPL32*	F: CCCACTCAGTATCAGCCAAA	101	79.28
		R: TTCTTCGCTTCGTAGTCTTGAG		
*Ubiquitin*	*Ubiq*	F: TGTCGTAAATGTTATGCACGTC	106	80.43
		R: TTACGTCTTGGGCTTCTTCTTT		
*Elongation factor 1α*	*EF1α*	F: TGTGGAGACTTTCACTGACTTC	116	77.50
		R: TGCCAGCAGACAGATCTTTAG		
*α-Tubulin*	*Tub*	F: CTGTCTGCATGCTTTCCAATAC	121	82.19
		R: TCCATACCTTCACCAACATACC		
*V-type proton ATPase catalytic subunit A*	*V-ATPase*	F: TTCAGGTCTTCTGGGGTTTG	132	81.0
		R: CGAAATCAGGAAAATGCCTCT		

*Mean efficiencies were calculated using Miner.

### Quantitative reverse transcription PCR

RT-qPCR was performed on 7500 Fast Real-Time PCR System (Thermo Scientific). Gene-specific primers (Table 2) were used in reactions (12uL), containing 6.5 μL of GoTaq® qPCR Master Mix (Promega), 3 μL of diluted cDNA template, 1.5 μL of ddH_2_O, and 0.5 μL of each specific primer (10 μM). The RT-qPCR program included an initial denaturation for 3 min at 95 °C followed by 40 cycles of 95 °C for 10 s, 60 °C for 30 s, and extension for 30 s at 72 °C. For melting curve analysis, a dissociation step cycle (55 °C for 10 s and then a gradual increase of 0.5 °C for 10 s until 95 °C) was added. Quantitative PCR reactions were carried out in 96-well plates with three technical replicates and three biological replicates. Three negative controls (no template) were included. The Cq values and PCR efficiency of each reaction were determined using Real-Time PCR Miner software^58^, a tool based on the kinetics of individual RT-qPCR reactions without the need of a standard curve. The arithmetic mean of amplification efficiencies of each primer pair was used for data normalization.

### Gene expression stability

The stability of the candidate reference genes was evaluated by algorithms GeNorm^19^, Normfinder^27^, BestKeeper^28^, and the comparative ΔCq method^26^. The seven genes analyzed were then compared and ranked the tested candidates based on a web-based analysis tool RefFinder (http://leonxie.esy.es/RefFinder/) that integrates the four methods. The algorithms GeNorm e NormFinder used in this study are included in the software GenEx version 6.0.1 (www.multid.se). The optimal number of reference genes for accurate normalization was calculated by pairwise variation using GeNorm in qBaseplus software (https://www.qbaseplus.com/).

The comparative ΔCq method establish a ranking based on pairwise of the genes by mean ΔCq values within a particular treatment and the stability of the gene is inversely proportional to its average standard deviation value (SD), and the gene with the lowest value is considered the most stable of Cq values between samples^26^. The GeNorm uses the pairwise variation between a specific candidate compared to the others to calculate an expression stability value (M-value). The lower the M-value, the higher stability of the gene, and an M value less than 1.05 is recommended^19^. The NormFinder calculates gene stability for all samples in any number of groups based on intra and inter group variations and combines values to ranking based on gene expression variation. The most stable gene will be the one with the lowest stability value (SV) according to the intra-group and inter-group variability of each gene^27^. Finally, the BestKeeper determines the most stably genes based on Pearson´s correlation coefficient for each gene, using raw data (Cq values) and PCR efficiency (E), with values closer to 1 indicating highest stability^28^.

### Validation of reference genes

Genes were evaluated comparatively as its performance in data normalization of RT-qPCR, using as target the expression levels of the *V-ATPase* in different development stages of *B. yothersi*. Five separate normalization factors (NFs) were calculated based on: i) a single reference gene with the lowest GEOMEAN value, ii) the geometric mean of the three most stable genes with the lowest GEOMEAN value (as determined by RefFinder) and iii) the geometric mean of two most unstable genes with the highest GEOMEAN value (determined by RefFinder). The relative quantification of the expression of V-ATPase normalized by each of these NFs was calculated using the derived 2^−ΔΔCq^ method^59^.

## Data Availability

The authors declare that the all data are availability.
